# EHMTI-0207. Abnormal brain excitability and cognitive dysfunction in adolescents with chronic daily headache

**DOI:** 10.1186/1129-2377-15-S1-E35

**Published:** 2014-09-18

**Authors:** A Sergeev, A Ratchin, T Avdeeva, G Tabeeva

**Affiliations:** 1 Department of Neurology, I.M. Sechenov First Moscow State Medical University, Moscow, Russia; 2 Department of Neurology, Smolensk State Medical Academy, Smolensk, Russia

## Introduction

People with daily chronic headache (CDH) commonly report impaired cognitive function. However, cognitive dysfunction in patients with CDH is poorly studied.

## Aims

We studied cognitive event related potential P300 elected by specific visual verbal and non-verbal stimuli “Headache” at adolescents with daily chronic headache (CDH).

## Methods

We recorded cognitive event related potential (ERP) P300 (significant stimuli - verbal (the word – «headache») and non-verbal (the image «headache»)) in 14 healthy adolescents and in 14 age, gender and socio-economic matched CDH patients. To test the ERP habituation, three consecutive blocks were recorded for P300 potential. Habituation of the ERPs P300 was defined as the % change of the N2/P3 amplitude between the 1st and 3rd block.

## Results

There was no difference in either the grant average N2/P3 latency or the grant average N2/P3 amplitudes as for verbal (323,8±70,8 ms; 19,6±5,5 mV and for the grant average latency and the amplitudes P300, respectively) as for non-verbal (342,4±64,2 ms; 21,2±6,1 mV for the grant average latency and the amplitudes P300 potential, respectively) stimuli at CDH group compared with controls.

During repeating stimulation (three blocks of stimuli) there was significant lack of habituation in CDH patients at specific verbal (+ 3,5%) and non-verbal stimuli (+0,8%) compared with controls (-15,6% and -13,4%, respectively).

## Conclusions

The lack of habituation in response to significant verbal and non-verbal stimuli points to the increase of relevance to cognitive function at CDH group. The results point at the development of memory and cognitive dysfunction in adolescents with CDH

No conflict of interest.

